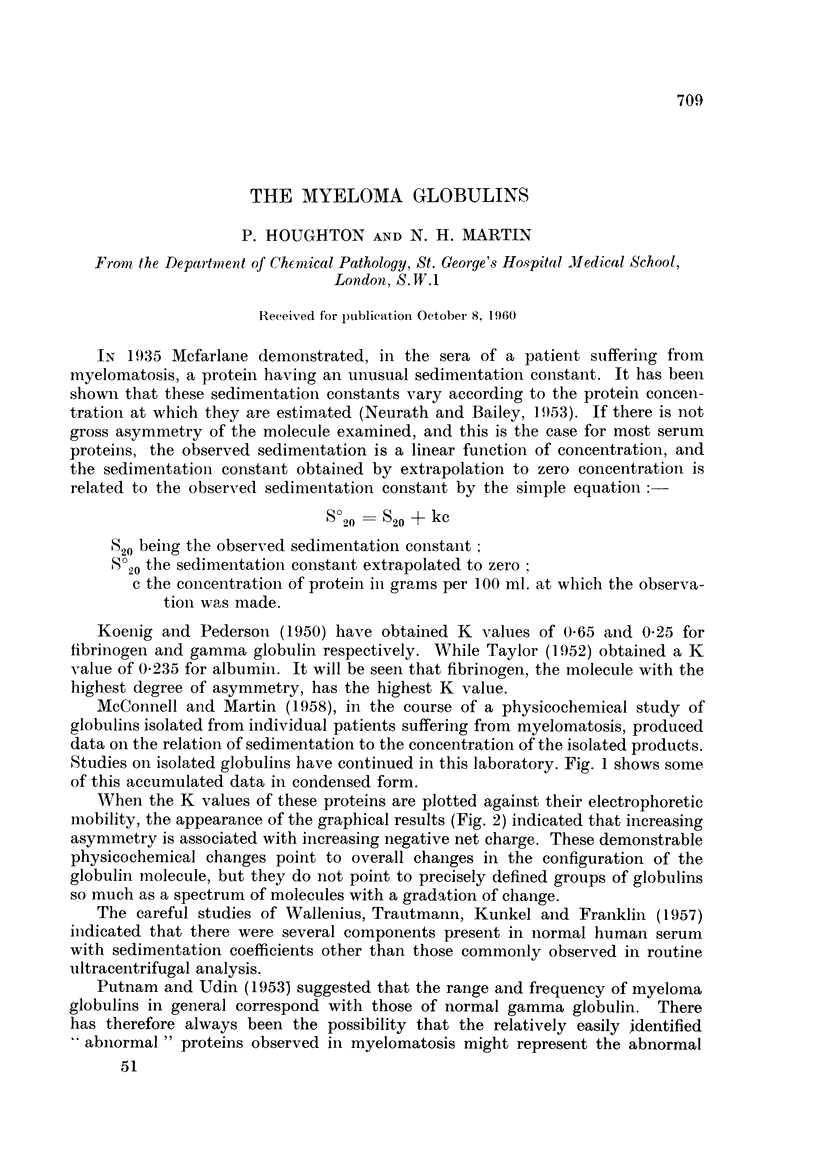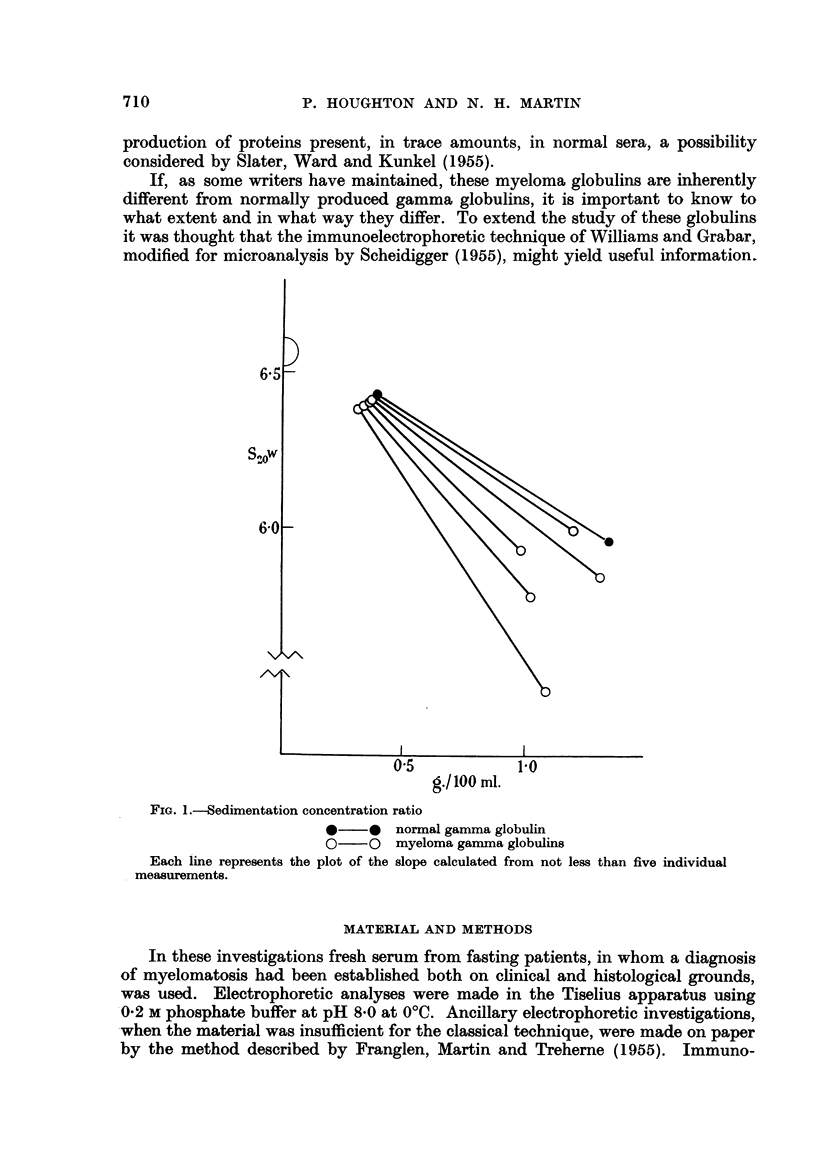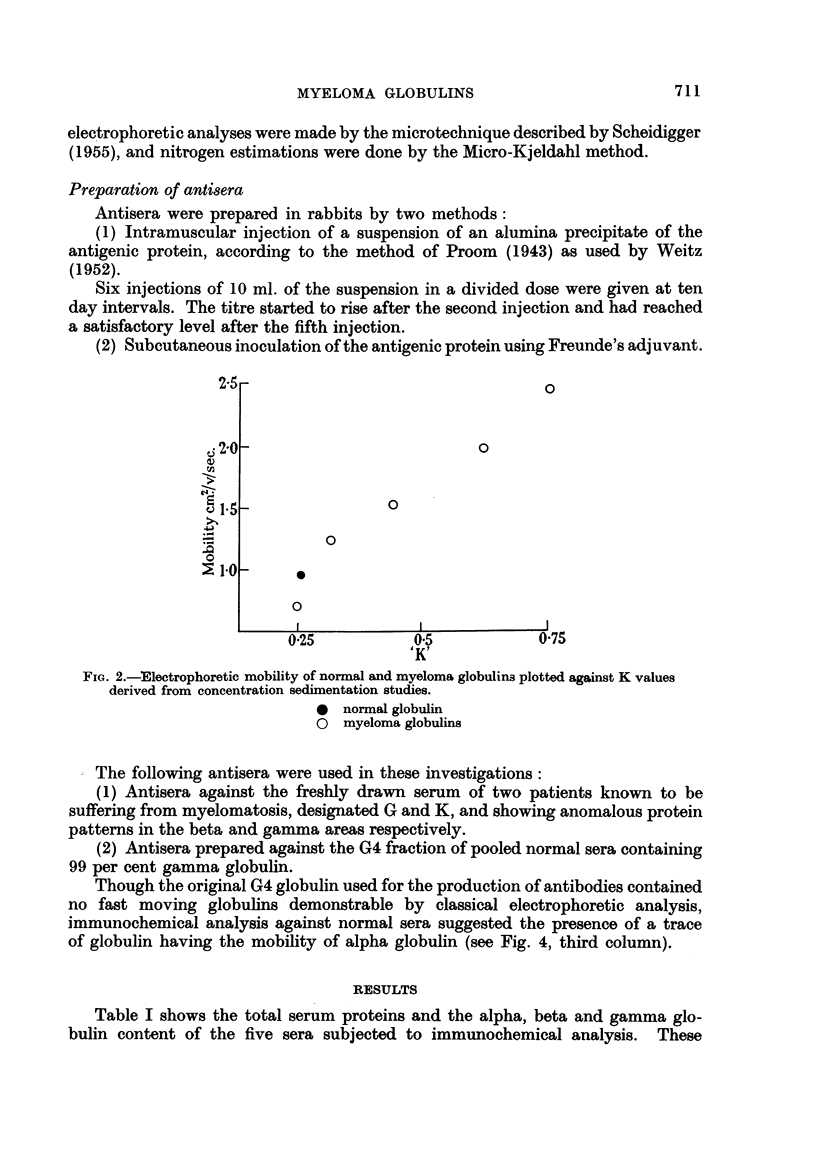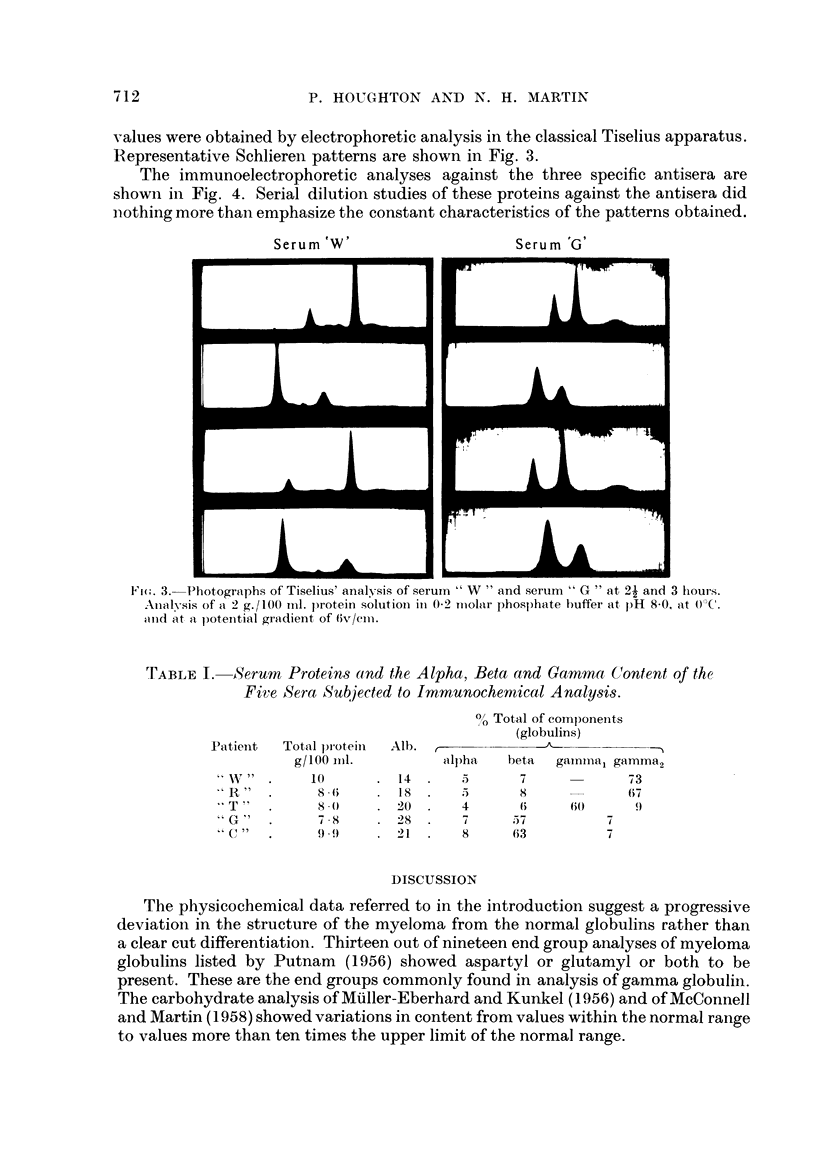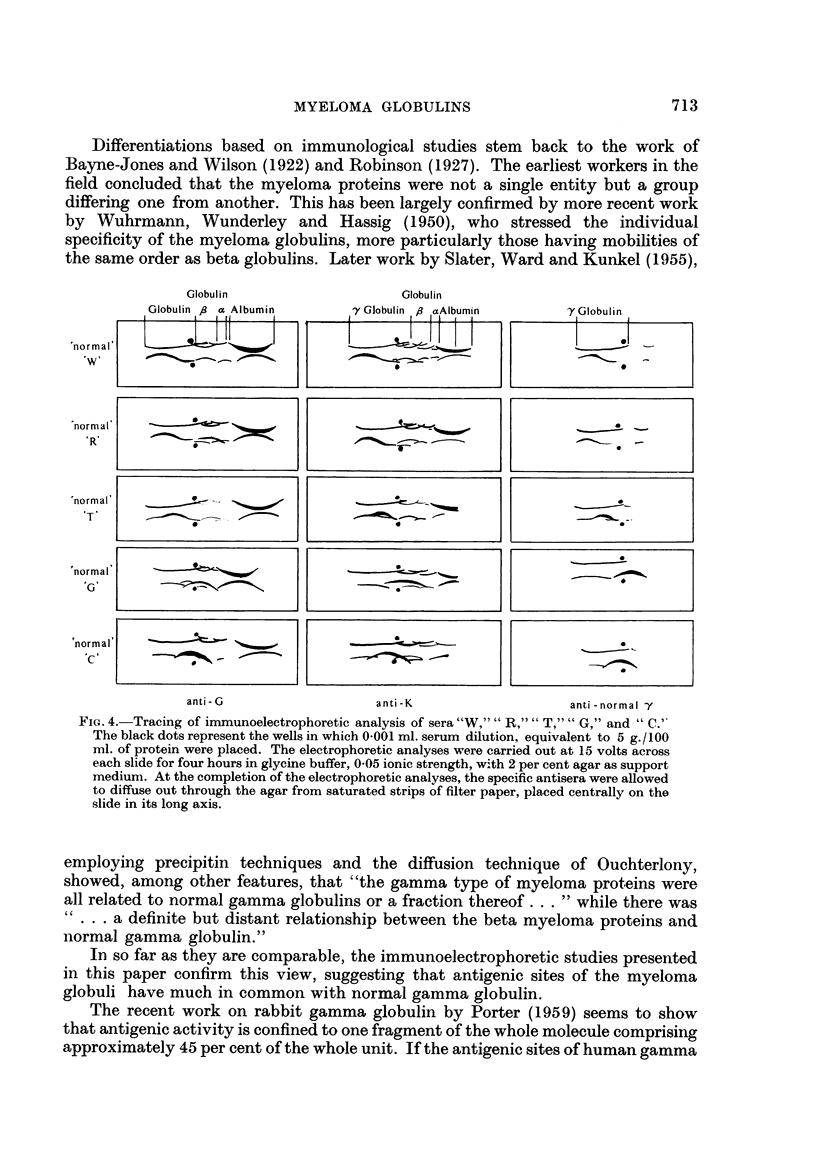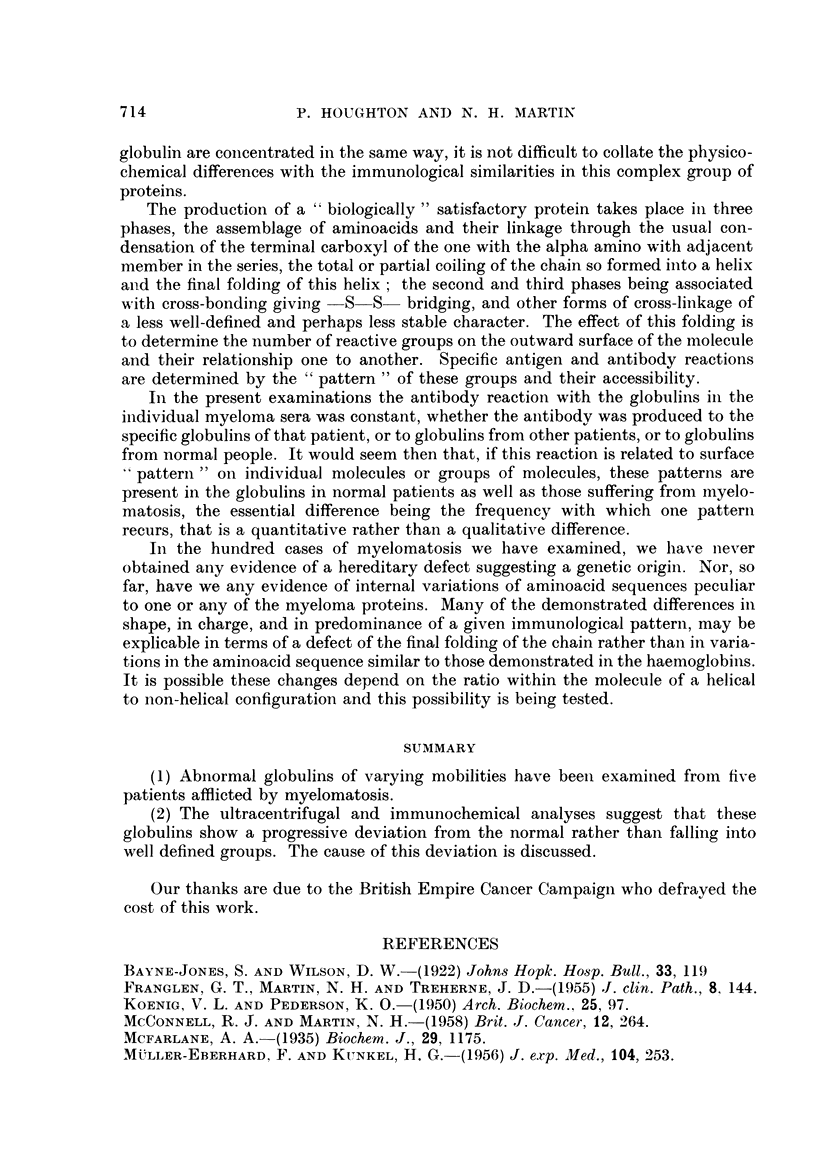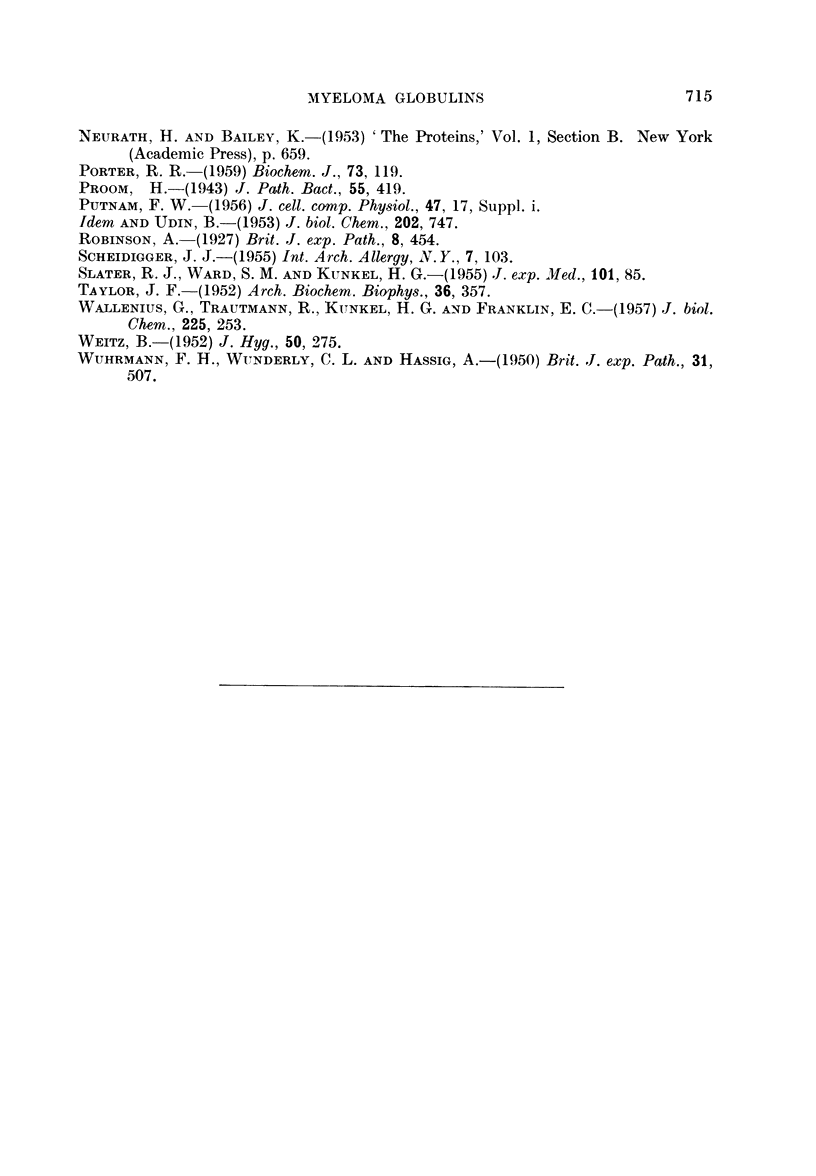# The Myeloma Globulins

**DOI:** 10.1038/bjc.1960.80

**Published:** 1960-12

**Authors:** P. Houghton, N. H. Martin


### THE MYELOMA GLOBULINS

P. HOUGHTON AND N. H. MARTIN

*From the Department of Chemical Pathology, St. George's Hospital Medical School, London, S.W.1*

Received for publication October 8, 1960

IN 1935 Mcfarlane demonstrated, in the sera of a patient suffering from myelomatosis, a protein having an unusual sedimentation constant. It has been shown that these sedimentation constants vary according to the protein concentration at which they are estimated (Neurath and Bailey, 1953). If there is not gross asymmetry of the molecule examined, and this is the case for most serum proteins, the observed sedimentation is a linear function of concentration, and the sedimentation constant obtained by extrapolation to zero concentration is related to the observed sedimentation constant by the simple equation :—

$$S^{\circ}_{20} = S_{20} + kc$$

$S_{20}$ being the observed sedimentation constant ;
$S^{\circ}_{20}$ the sedimentation constant extrapolated to zero ;
c the concentration of protein in grams per 100 ml. at which the observation was made.

Koenig and Pederson (1950) have obtained K values of 0·65 and 0·25 for fibrinogen and gamma globulin respectively. While Taylor (1952) obtained a K value of 0·235 for albumin. It will be seen that fibrinogen, the molecule with the highest degree of asymmetry, has the highest K value.

McConnell and Martin (1958), in the course of a physicochemical study of globulins isolated from individual patients suffering from myelomatosis, produced data on the relation of sedimentation to the concentration of the isolated products. Studies on isolated globulins have continued in this laboratory. Fig. 1 shows some of this accumulated data in condensed form.

When the K values of these proteins are plotted against their electrophoretic mobility, the appearance of the graphical results (Fig. 2) indicated that increasing asymmetry is associated with increasing negative net charge. These demonstrable physicochemical changes point to overall changes in the configuration of the globulin molecule, but they do not point to precisely defined groups of globulins so much as a spectrum of molecules with a gradation of change.

The careful studies of Wallenius, Trautmann, Kunkel and Franklin (1957) indicated that there were several components present in normal human serum with sedimentation coefficients other than those commonly observed in routine ultracentrifugal analysis.

Putnam and Udin (1953) suggested that the range and frequency of myeloma globulins in general correspond with those of normal gamma globulin. There has therefore always been the possibility that the relatively easily identified "abnormal" proteins observed in myelomatosis might represent the abnormal production of proteins present, in trace amounts, in normal sera, a possibility considered by Slater, Ward and Kunkel (1955).

If, as some writers have maintained, these myeloma globulins are inherently different from normally produced gamma globulins, it is important to know to what extent and in what way they differ. To extend the study of these globulins it was thought that the immunoelectrophoretic technique of Williams and Grabar, modified for microanalysis by Scheidigger (1955), might yield useful information.

FIG. 1.—Sedimentation concentration ratio

●——● normal gamma globulin
○——○ myeloma gamma globulins

Each line represents the plot of the slope calculated from not less than five individual measurements.

### MATERIAL AND METHODS

In these investigations fresh serum from fasting patients, in whom a diagnosis of myelomatosis had been established both on clinical and histological grounds, was used. Electrophoretic analyses were made in the Tiselius apparatus using 0·2 M phosphate buffer at pH 8·0 at 0°C. Ancillary electrophoretic investigations, when the material was insufficient for the classical technique, were made on paper by the method described by Franglen, Martin and Treherne (1955). Immunoelectrophoretic analyses were made by the microtechnique described by Scheidigger (1955), and nitrogen estimations were done by the Micro-Kjeldahl method.

*Preparation of antisera*

Antisera were prepared in rabbits by two methods :

(1) Intramuscular injection of a suspension of an alumina precipitate of the antigenic protein, according to the method of Proom (1943) as used by Weitz (1952).

Six injections of 10 ml. of the suspension in a divided dose were given at ten day intervals. The titre started to rise after the second injection and had reached a satisfactory level after the fifth injection.

(2) Subcutaneous inoculation of the antigenic protein using Freunde's adjuvant.

FIG. 2.—Electrophoretic mobility of normal and myeloma globulins plotted against K values derived from concentration sedimentation studies.

● normal globulin
○ myeloma globulins

The following antisera were used in these investigations :

(1) Antisera against the freshly drawn serum of two patients known to be suffering from myelomatosis, designated G and K, and showing anomalous protein patterns in the beta and gamma areas respectively.

(2) Antisera prepared against the G4 fraction of pooled normal sera containing 99 per cent gamma globulin.

Though the original G4 globulin used for the production of antibodies contained no fast moving globulins demonstrable by classical electrophoretic analysis, immunochemical analysis against normal sera suggested the presence of a trace of globulin having the mobility of alpha globulin (see Fig. 4, third column).

### RESULTS

Table I shows the total serum proteins and the alpha, beta and gamma globulin content of the five sera subjected to immunochemical analysis. These values were obtained by electrophoretic analysis in the classical Tiselius apparatus. Representative Schlieren patterns are shown in Fig. 3.

The immunoelectrophoretic analyses against the three specific antisera are shown in Fig. 4. Serial dilution studies of these proteins against the antisera did nothing more than emphasize the constant characteristics of the patterns obtained.

FIG. 3.—Photographs of Tiselius' analysis of serum " W " and serum " G " at 2½ and 3 hours. Analysis of a 2 g./100 ml. protein solution in 0·2 molar phosphate buffer at pH 8·0, at 0°C. and at a potential gradient of 6v/cm.

TABLE I.—*Serum Proteins and the Alpha, Beta and Gamma Content of the Five Sera Subjected to Immunochemical Analysis.*

### DISCUSSION

The physicochemical data referred to in the introduction suggest a progressive deviation in the structure of the myeloma from the normal globulins rather than a clear cut differentiation. Thirteen out of nineteen end group analyses of myeloma globulins listed by Putnam (1956) showed aspartyl or glutamyl or both to be present. These are the end groups commonly found in analysis of gamma globulin. The carbohydrate analysis of Müller-Eberhard and Kunkel (1956) and of McConnell and Martin (1958) showed variations in content from values within the normal range to values more than ten times the upper limit of the normal range.

Differentiations based on immunological studies stem back to the work of Bayne-Jones and Wilson (1922) and Robinson (1927). The earliest workers in the field concluded that the myeloma proteins were not a single entity but a group differing one from another. This has been largely confirmed by more recent work by Wuhrmann, Wunderley and Hassig (1950), who stressed the individual specificity of the myeloma globulins, more particularly those having mobilities of the same order as beta globulins. Later work by Slater, Ward and Kunkel (1955),

FIG. 4.—Tracing of immunoelectrophoretic analysis of sera "W," " R," " T," " G," and " C." The black dots represent the wells in which 0·001 ml. serum dilution, equivalent to 5 g./100 ml. of protein were placed. The electrophoretic analyses were carried out at 15 volts across each slide for four hours in glycine buffer, 0·05 ionic strength, with 2 per cent agar as support medium. At the completion of the electrophoretic analyses, the specific antisera were allowed to diffuse out through the agar from saturated strips of filter paper, placed centrally on the slide in its long axis.

employing precipitin techniques and the diffusion technique of Ouchterlony, showed, among other features, that "the gamma type of myeloma proteins were all related to normal gamma globulins or a fraction thereof . . . " while there was " . . . a definite but distant relationship between the beta myeloma proteins and normal gamma globulin."

In so far as they are comparable, the immunoelectrophoretic studies presented in this paper confirm this view, suggesting that antigenic sites of the myeloma globuli have much in common with normal gamma globulin.

The recent work on rabbit gamma globulin by Porter (1959) seems to show that antigenic activity is confined to one fragment of the whole molecule comprising approximately 45 per cent of the whole unit. If the antigenic sites of human gamma globulin are concentrated in the same way, it is not difficult to collate the physico-chemical differences with the immunological similarities in this complex group of proteins.

The production of a " biologically " satisfactory protein takes place in three phases, the assemblage of aminoacids and their linkage through the usual condensation of the terminal carboxyl of the one with the alpha amino with adjacent member in the series, the total or partial coiling of the chain so formed into a helix and the final folding of this helix ; the second and third phases being associated with cross-bonding giving —S—S— bridging, and other forms of cross-linkage of a less well-defined and perhaps less stable character. The effect of this folding is to determine the number of reactive groups on the outward surface of the molecule and their relationship one to another. Specific antigen and antibody reactions are determined by the " pattern " of these groups and their accessibility.

In the present examinations the antibody reaction with the globulins in the individual myeloma sera was constant, whether the antibody was produced to the specific globulins of that patient, or to globulins from other patients, or to globulins from normal people. It would seem then that, if this reaction is related to surface " pattern " on individual molecules or groups of molecules, these patterns are present in the globulins in normal patients as well as those suffering from myelomatosis, the essential difference being the frequency with which one pattern recurs, that is a quantitative rather than a qualitative difference.

In the hundred cases of myelomatosis we have examined, we have never obtained any evidence of a hereditary defect suggesting a genetic origin. Nor, so far, have we any evidence of internal variations of aminoacid sequences peculiar to one or any of the myeloma proteins. Many of the demonstrated differences in shape, in charge, and in predominance of a given immunological pattern, may be explicable in terms of a defect of the final folding of the chain rather than in variations in the aminoacid sequence similar to those demonstrated in the haemoglobins. It is possible these changes depend on the ratio within the molecule of a helical to non-helical configuration and this possibility is being tested.

### SUMMARY

(1) Abnormal globulins of varying mobilities have been examined from five patients afflicted by myelomatosis.

(2) The ultracentrifugal and immunochemical analyses suggest that these globulins show a progressive deviation from the normal rather than falling into well defined groups. The cause of this deviation is discussed.

Our thanks are due to the British Empire Cancer Campaign who defrayed the cost of this work.

### REFERENCES

Bayne-Jones, S. and Wilson, D. W.—(1922) *Johns Hopk. Hosp. Bull.*, **33**, 119
Franglen, G. T., Martin, N. H. and Treherne, J. D.—(1955) *J. clin. Path.*, **8**, 144.
Koenig, V. L. and Pederson, K. O.—(1950) *Arch. Biochem.*, **25**, 97.
McConnell, R. J. and Martin, N. H.—(1958) *Brit. J. Cancer*, **12**, 264.
Mcfarlane, A. A.—(1935) *Biochem. J.*, **29**, 1175.
Müller-Eberhard, F. and Kunkel, H. G.—(1956) *J. exp. Med.*, **104**, 253.

NEURATH, H. AND BAILEY, K.—(1953) 'The Proteins,' Vol. 1, Section B. New York (Academic Press), p. 659.
PORTER, R. R.—(1959) *Biochem. J.*, **73**, 119.
PROOM, H.—(1943) *J. Path. Bact.*, **55**, 419.
PUTNAM, F. W.—(1956) *J. cell. comp. Physiol.*, **47**, 17, Suppl. i.
*Idem* AND UDIN, B.—(1953) *J. biol. Chem.*, **202**, 747.
ROBINSON, A.—(1927) *Brit. J. exp. Path.*, **8**, 454.
SCHEIDIGGER, J. J.—(1955) *Int. Arch. Allergy, N.Y.*, **7**, 103.
SLATER, R. J., WARD, S. M. AND KUNKEL, H. G.—(1955) *J. exp. Med.*, **101**, 85.
TAYLOR, J. F.—(1952) *Arch. Biochem. Biophys.*, **36**, 357.
WALLENIUS, G., TRAUTMANN, R., KUNKEL, H. G. AND FRANKLIN, E. C.—(1957) *J. biol. Chem.*, **225**, 253.
WEITZ, B.—(1952) *J. Hyg.*, **50**, 275.
WUHRMANN, F. H., WUNDERLY, C. L. AND HASSIG, A.—(1950) *Brit. J. exp. Path.*, **31**, 507.